# [^99m^Tc]Tc-hydroxydiphosphonate uptake in soft tissue is associated with amyloid load in subcutaneous abdominal fat tissue and mortality in wild-type transthyretin amyloidosis patients

**DOI:** 10.1007/s00259-024-06865-w

**Published:** 2024-08-08

**Authors:** Hendrea Sanne Aletta Tingen, Dion Groothof, Alwin Tubben, Johan Bijzet, Ewout J. Houwerzijl, Friso L. H. Muntinghe, Paul A. van der Zwaag, Peter van der Meer, Bouke P. C. Hazenberg, Riemer H. J. A. Slart, Hans L. A. Nienhuis

**Affiliations:** 1 Department of Nuclear Medicine and Molecular Imaging, Groningen Amyloidosis Center of Expertise, University Medical Center Groningen, Hanzeplein 1, 9713GZ Groningen, The Netherlands; 2 Department of Internal Medicine, Groningen Amyloidosis Center of Expertise, University Medical Center Groningen, Hanzeplein 1, 9713GZ, Groningen, The Netherlands; 3 Department of Cardiology, Groningen Amyloidosis Center of Expertise, University Medical Center Groningen, Hanzeplein 1, 9713GZ Groningen, The Netherlands; 4 Department of Laboratory Medicine, Groningen Amyloidosis Center of Expertise, University Medical Center Groningen, Hanzeplein 1, 9713GZ Groningen, The Netherlands; 5 Department of Clinical Genetics, Groningen Amyloidosis Center of Expertise, University Medical Center Groningen, Hanzeplein 1, 9713GZ Groningen, The Netherlands; 6 Department of Rheumatology & Clinical Immunology, Groningen Amyloidosis Center of Expertise, University Medical Center Groningen, Hanzeplein 1 9713GZ Groningen, The Netherlands; 7 Biomedical Photonic Imaging Group, Faculty of Science and Technology, University of Twente, Enschede, The Netherlands

**Keywords:** ATTR, Bone scintigraphy, Cardiac amyloidosis, Extracardiac, Survival

## Abstract

**Purpose:**

Bone scintigraphy is key to non-invasively diagnosing wild-type transthyretin (ATTRwt) amyloidosis, and is mainly used to assess cardiac radiotracer uptake. However, extracardiac radiotracer uptake is also observed. We investigated whether intensity of soft tissue radiotracer uptake is associated with amyloid load in subcutaneous abdominal fat tissue and with mortality.

**Methods:**

This prospective cohort study included 94 ATTRwt amyloidosis patients and 26 amyloid-negative heart failure controls who underwent whole-body [^99m^Tc]Tc-hydroxydiphosphonate scintigraphy. Site-to-background ratios were calculated for heart, elbows, subcutaneous tissue, shoulders and wrists on anterior planar bone scintigraphy images using rib and whole-body radiotracer uptake as background. Fat tissue aspirates were stained with Congo red to grade amyloid load. Site-to-rib ratios were compared between ATTRwt amyloidosis patients and controls, and associations of site-to-background ratio with Congo red score and all-cause mortality were studied.

**Results:**

ATTRwt amyloidosis patients had higher soft tissue-to-rib, heart-to-rib and heart-to-whole body ratios compared with controls. The intensity of soft tissue uptake was positively associated with amyloid load in fat tissue in ATTRwt amyloidosis patients. Estimated glomerular filtration rate, N-terminal brain natriuretic propeptide, high-sensitivity cardiac troponin T (hs-cTnT), and the prognostic Mayo and NAC staging system were associated with all-cause mortality in univariable models. Soft tissue/rib ratio, hs-cTnT and the prognostic staging systems were the only two variables that were independently associated withall-cause mortality.

**Conclusion:**

Soft tissue radiotracer uptake on bone scintigraphy in ATTRwt amyloidosis patients is positively associated with amyloid load in abdominal fat tissue and is independently associated with mortality.

**Supplementary Information:**

The online version contains supplementary material available at 10.1007/s00259-024-06865-w.

## Introduction

Wild-type transthyretin (ATTRwt) amyloidosis is a progressive protein-misfolding disease characterized by deposition of amyloid predominantly in ligaments, tendons and the myocardium, causing carpal tunnel syndrome, spinal stenosis and ATTR cardiomyopathy (ATTR-CM) [1, 2].

Bone scintigraphy is the cornerstone imaging modality to non-invasively diagnose ATTR-CM [3, 4]. The calcium-binding radiotracers used in bone scintigraphy are assumed to bind to microcalcifications in the vicinity of ATTR amyloid deposits, thereby indicating the location of ATTR amyloid deposits with high diagnostic accuracy. A recent study shows that cardiac radiotracer uptake on bone scintigraphy is associated with histological amyloid load in myocardial tissue [5].

Although bone scintigraphy is mainly used to detect ATTR-CM, radiotracer uptake in extracardiac tissue can also be noted in ATTRwt amyloidosis patients [6, 7]. There is some evidence that extracardiac radiotracer uptake corresponds with histopathologically-confirmed amyloid deposits in subcutaneous abdominal fat tissue and several organs but whether intensity of extracardiac radiotracer uptake is associated with fat tissue and organ amyloid load has not been investigated [8, 9].

The presence of cardiac radiotracer uptake on bone scintigraphy is associated with worse survival, and additional presence of extracardiac radiotracer uptake is associated with even poorer survival [6, 7]. When taking into account not merely the presence of radiotracer uptake but also the intensity of radiotracer uptake, conflicting results have been reported regarding the prognostic value of the intensity of cardiac radiotracer uptake [6, 10–12] and the prognostic value of the intensity of extracardiac radiotracer uptake is unknown.

Therefore, the objective of this cohort study was to investigate whether intensity of radiotracer uptake in soft tissue reflects amyloid load as measured by subcutaneous abdominal fat tissue aspirates and whether the intensity of myocardial radiotracer uptake on bone scintigraphy and at various extracardiac sites is associated with mortality.

## Methods

### Study population

In this prospective cohort study, all new consecutive ATTRwt amyloidosis patients who underwent a whole-body bone scintigraphy at the National Amyloidosis Centre of Expertise of the University Medical Center Groningen in the Netherlands between 2012 and 2023 were enrolled. Diagnosis of ATTRwt amyloidosis was established by Perugini grade 2 or 3 cardiac radiotracer uptake on bone scintigraphy and proven amyloid in fat biopsy in individuals with heart failure with preserved ejection fraction according to the ESC consensus criteria [13]. Immunoglobulin light chain amyloidosis was excluded by blood and urine testing and hereditary ATTR amyloidosis was excluded by gene panel testing in all individuals. Additionally, 26 amyloid-negative heart failure patients enrolled in the VIP-HF study [14] and who had undergone bone scintigraphy were included as control population.

### Collection of data

Sociodemographic characteristics, information about treatment, estimated glomerular filtration rate (eGFR), serum levels of high-sensitivity cardiac troponin T (hs-cTnT) and N-terminal pro-brain-type natriuretic peptide (NT-proBNP), and left ventricular ejection fraction (LVEF) on echocardiographywere extracted from the patient records. Laboratory tests were performed as part of routine clinical care and according to clinical standards. Additionally, information regarding all-cause mortality and cardiac mortality as of May 2024 was obtained.

### Amyloid load in fat tissue

Subcutaneous abdominal fat aspirates were obtained and processed as described previously [15]. All fat smears were assessed blinded for clinical data and graded by two experienced and independent observers. A validated semiquantitative grading system was used, ranging from 0 to 4 + : 0 = negative; 1 +  = minute, < 1% of the surface area; 2 +  = little, between 1 and 10%; 3 +  = moderate, between 10 and 60%; and 4 +  = abundant, > 60% [16].

### Bone scintigraphy

All patients received 450–750 MBq [^99m^Tc]Tc-hydroxydiphosphonate (HDP) intravenously. Bone scintigraphy was performed on dedicated single photon emission computed tomography (SPECT)/CT systems (Symbia T2, Symbia T16 or Symbia Intevo, Siemens Healthineers, Erlangen, Germany) equipped with a low-energy high-resolution collimator. Planar whole-body scans were obtained three hours after radiotracer injection from posterior and anterior view. Acquisition time was 12 min in total, and 6 min per view. SPECT/CT of the thorax were acquired using a 180° configuration, 64 views, 20 s per view and a 128 × 128 matrix.

**Fig. 1:**
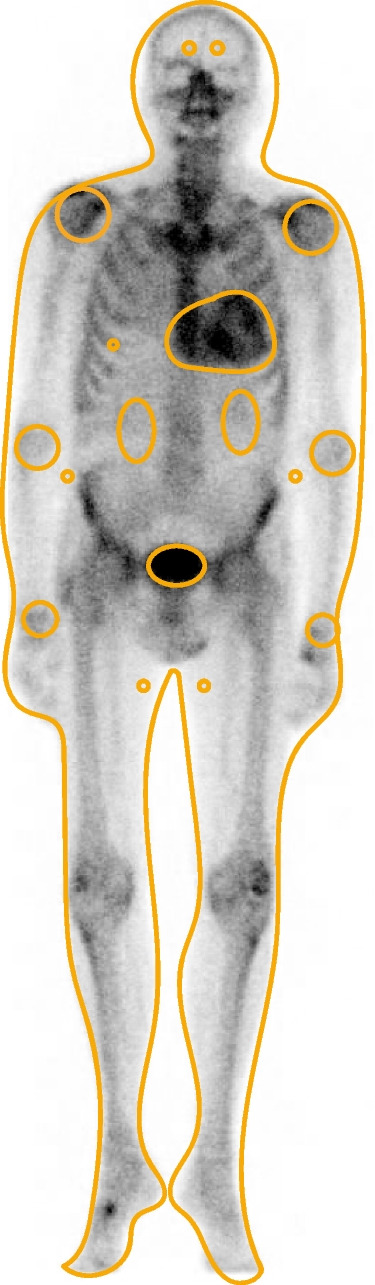
Representation of regions of interest drawing method for calculation of ratios

Planar images were compared with SPECT images to verify that radiotracer uptake was in the myocardium and not in the blood pool. Bone scintigraphies were scored by two independent observers. Regions of interest (ROI) were placed over the heart (free shape), the sixth right rib (round), both shoulders (round), both elbows (round), both wrists (round), abdominal soft tissue on both lateral sides of the torso (round), thigh soft tissue proximally on the medial side of both legs (round), both kidneys (oval), the bladder (free shape) and around the outline of the body (free shape) on the anterior planar images (Fig. 1). The size of the ROIs depended on the anatomy of individual patients. If increased uptake due to extravasation at the site of the radiotracer administration was present within the intended elbow ROI, no ROI was placed. Mean radiotracer uptake in all soft tissue ROIs and in bilateral regions were averaged to create one estimation for each site. Mean radiotracer uptake at all sites was divided by the mean uptake in the sixth right rib to standardize outcomes to allow for interpatient comparison. Cardiac radiotracer uptake was additionally expressed as heart-to-whole body (H/WB) ratio in individuals with cardiac radiotracer uptake and was calculated by dividing cardiac uptake by the whole body uptake corrected for uptake in kidneys and bladder [17]. In individuals without cardiac radiotracer uptake, a circular ROI was placed parasternally at the left side of the thorax between the sternum and the ribs and used in the calculation of the H/WB ratio.

### Statistics

Clinical characteristics are reported as median [25th and 75th percentile] and counts (percentage) for continuous and categorical data, respectively. Differences in radiotracer uptake between ATTRwt amyloidosis patients and controls were evaluated with Mann–Whitney U tests.

Jonckheere-Terpstra tests were used to evaluate radiotracer uptake across increasing grades of Congo red-stained fat tissue samples in ATTRwt amyloidosis patients. Controls were excluded from this analysis. Due to a low number of patients with Congo red grade > 2 + , patients having grade 2 + , 3 + or 4 + were pooled into a single group to maintain statistical power. Homogeneity of variance was assessed and confirmed in both groups and across increasing Congo red grades and confirmed for all site-to-rib ratios and H/WB ratio.

Effects of site-to-rib ratios on the hazard of mortality were quantified with Cox proportional hazards models in ATTRwt amyloidosis patients. Controls were excluded from this analysis. Age, sex, all ratios on bone scintigraphy, the UK National Amyloidosis Centre (NAC) staging system [18] and the Mayo staging system [19] for cardiac ATTR amyloidosis, eGFR, NT-proBNP, hs-cTnT, and LVEF on echocardiography were first assessed in a univariable model. Hazard ratios (HR) and 95% confidence interval (CI) were calculated for each variable. Variables with a significant association with mortality in the univariable model were added to a multivariable model also containing soft tissue/rib ratio and which was corrected for age, to assess which variables were independently associated with mortality. Additionally, soft tissue/rib ratio was added to the two prognostic staging systems in a multivariable regression analysis corrected for age. All survival analyses were conducted for both all-cause mortality and cardiac mortality. The proportional hazards assumption was assessed by inspecting Schoenfeld residuals and confirmed for all time-to-event analyses. Median follow-up duration for survival analysis was calculated by reverse Kaplan Meier method [20].

A two-sided *p* < 0.05 was considered to indicate statistical significance. Multiple testing was accounted for using the Bonferroni method. Intraclass correlation coefficient (ICC) was used to assess interobserver agreement for Congo red score in fat tissue and ratios on bone scintigraphy. Statistical analyses were performed using SPSS version 28 software (IBM Corp, Armonk, New York, USA).

### Ethical approval

All procedures were in compliance with the Declaration of Helsinki. The study was approved by the institutional review board of the University Medical Center Groningen and requirement for consent was waived (Registration number: 202100405).

## Results

### Clinical and demographic characteristics

In total, 198 ATTRwt amyloidosis patients were referred to our centre between 2012 and 2023. 94 of these patients had undergone whole-body bone scintigraphy at our centre and were included in this study. Clinical characteristics of the 94 ATTRwt amyloidosis patients and 26 control patients are listed in Table 1. ATTRwt amyloidosis patients were significantly older of age and had higher circulating hs-cTnT and NT-proBNP levels compared to control patients. SimplePara>

**Table 1:** Patient characteristics and bone scintigraphy at baseline

Characteristic	ATTRwt	Control	*P*-value
Number of patients	94	26	
Age (years)	75 [72–80]	72 [68–75]	.014*
Male	88 (42%)	12 (46%)	< .001*
Grade of Congo red-stained fat tissue		N/A	N/A
0 + 1 + 2 + 3 + 4 +	35 (37%) 37 (39%) 10 (11%) 11 (12%) 1 (1%)		
Serum biomarkers			
hs-cTnT (ng/L) NT-proBNP (ng/L) eGFR (ml/min*1.73m^2^)	54 [40–72] 2877 [1642–3921] 60 [47–70]	19 [14−26] 710 [444–2728] 53 [40–71]	< .001* < .001* .293
NAC staging		N/A	NA
Stage 1 Stage 2 Stage 3	48 (51%) 30 (32%) 16 (17%)		
Mayo staging		N/A	NA
Stage 1 Stage 2 Stage 3	32 (32%) 34 (34%) 33 (33%)		
Bone scintigraphy			
Heart/rib ratio Heart/WB ratio Elbow/rib ratio Soft tissue/rib ratio Shoulder/rib ratio Wrist/rib ratio	1.92 [1.59–2.32] 6.06 [5.38–6.79] 0.62 [0.49–0.76] 0.31 [0.24–0.39] 1.20 [1.02–1.38] 0.64 [0.53–0.86]	0.87 [0.71–0.96] 1.83 [1.60–2.01] 0.71 [0.55–0.99] 0.26 [0.22–0.29] 1.29 [1.12–1.48] 0.82 [0.54–0.94]	< .001* < .001* .316 .021* > .999 > .999
Perugini score:			< .001*
0 1 2 3	0 (0%) 0 (0%) 39 (41%) 55 (59%)	26 (100%) 0 (0%) 0 (0%) 0 (0%)	

Values are median [interquartile range] or number of patients (percentage)

*ATTRwt* wild type ATTR amyloidosis, *hs-cTnT* high sensitivity cardiac troponin T, *NT-proBNP* N-terminal pro-brain-type natriuretic peptide, *eGFR* estimated glomerular filtration rate, * = significant difference,* NA* not applicable

### Comparison of radiotracer uptake on bone scintigraphy between ATTRwt amyloidosis patients and control patients

**Fig. 2:**
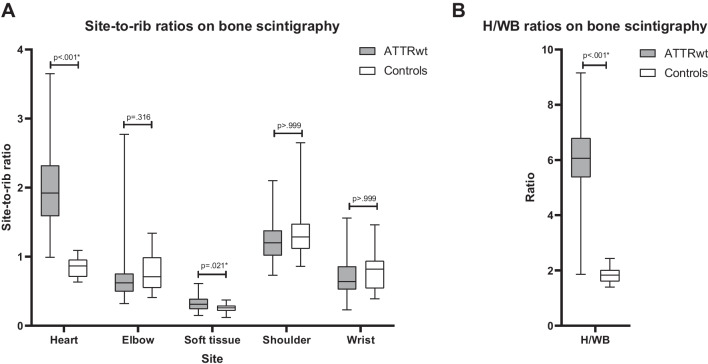
Distribution of ratios of bone radiotracer uptake per site in ATTRwt amyloidosis patients versus control patients. Boxplots of site-to-rib ratio (**A**) and H/WB ratio (**B**) of bone radiotracer uptake per site in ATTRwt amyloidosis patients versus control patients. The horizontal line within the box represents the group median and the box represents the interquartile range. Whiskers represent the total range. Bonferroni corrected *p*-values are presented. ATTRwt = wild type transthyretin amyloid, H/WB = Heart-to-whole body, * = significant difference

Interobserver agreement for ratios on bone scintigraphy was 0.75 (95% (CI): 0.57–0.85, *p* < 0.001*), indicating good agreement between the two observers. Results from bone scintigraphy are presented in Table 1 and Fig. 2. In ATTRwt amyloidosis patients, soft tissue/rib, heart/rib and H/WB ratios were higher compared with non-amyloid heart failure patients (all *p* < 0.05). No between-group difference was found for elbow/rib, shoulder/rib and wrist/rib ratios (Fig. 2).

### Radiotracer uptake on bone scintigraphy by Congo red grade in subcutaneous abdominal fat tissue aspirates in ATTRwt amyloidosis patients

**Fig. 3:**
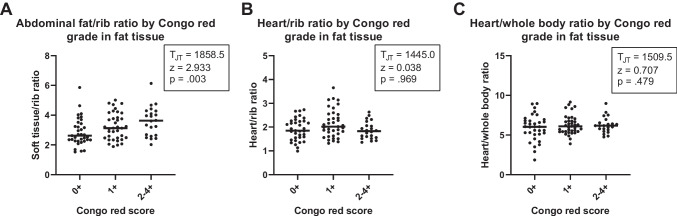
Distributions of soft tissue/rib, heart/rib and H/WB ratios of bone radiotracer uptake in ATTRwt amyloidosis patients according to grading of Congo red-stained fat tissue. Scatter plots show the ratios on bone scintigraphies for ATTRwt amyloidosis patients with different grades of Congo red-stained fat tissues. The bold horizontal line represents the group median. **A** Soft tissue/rib ratio. **B** Heart/rib ratio. **C** H/WB ratio. ATTRwt = wild type transthyretin amyloid, H/WB = Heart-to-whole body, * = significant difference

Interobserver agreement for Congo red scores of fat tissue was 0.90 (95% CI: 0.82–0.97, *p* < 0.001*), indicating good agreement between the two observers. Higher grades of Congo red-stained fat tissue samples were associated with higher median soft tissue/rib but not with higher heart/rib and H/WB ratios in ATTRwt amyloidosis patients (Fig. 3).

### Associations between ratios on bone scintigraphy and mortality in ATTRwt amyloidosis patients

**Fig. 4:**
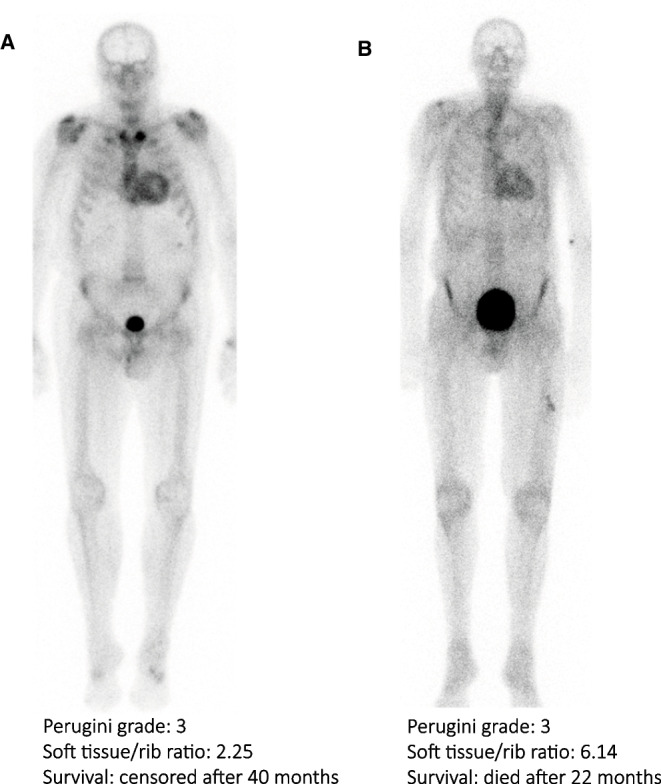
Example of a patient with a low soft tissue/rib ratio (**A**) and a patient with high soft tissue/rib ratio. Both patients had Perugini grade 3 cardiac radiotracer uptake on bone scintigraphy. Patient A exhibited a soft tissue/rib ratio of 2.25 and the patient was alive at the end of follow-up at 40 months after bone scintigraphy. Patient B exhibited a soft tissue/rib ratio of 6.14 and died after 22 months of follow-up

Median follow-up duration of all included ATTRwt amyloidosis patients was 38 [17–55] months. During this follow-up, a total of 48 (51%) deaths were registered after a median of 55 (95% CI 52–59) months. In univariable time-to-event analysis, higher NT-proBNP levels, higher hs-cTnT levels, and higher NAC and Mayo stages were associated with increased risk of all-cause mortality (Table 2). Conversely, higher estimated glomerular filtration rate was associated with lower risk of all-cause mortality (Table 2). These variables, except for the NAC and Mayo stages, were entered in a multivariable model that additionally included soft tissue/rib ratio and age. Only soft tissue/rib ratio and hs-cTnT were independent predictors of all-cause mortality. eGFR and NT-proBNP were not significant independent predictors of all-cause mortality in ATTRwt amyloidosis patients (Table 2). An example of a patient with low and a patient with high soft tissue/rib ratio is provided in Fig. 4.

**Table 2:** Univariable and multivariable cox proportional hazards regression analysis for all-cause mortality in ATTRwt amyloidosis patients

	Univariable analysis			Multivariable analysis		
Variable	HR	95% CI	*p*-value	HR	95% CI	*p*-value
Age (per year)	1.02	0.97–1.07	.407	1.03	0.97–1.09	.374
Sex (male)	0.45	0.16–1.26	.129			●
Congo red grade			.643			●
1 + vs 0 + > 2 + vs 0 +	1.27 0.92	0.66–2.44 0.41–2.04	.482 .831			
Perugini score (grade 2)	0.96	0.54–1.71	.882			●
eGFR (per ml/min*1.73m^2^)	0.98	0.96–0.99	.010*	0.99	0.97–1.01	.235
NT-proBNP (per 100 ng/L)	1.01	1.01–1.02	< .001*	1.01	1.00–1.01	.122
Hs-cTnT (per 1 ng/L)	1.02	1.01–1.03	< .001*	1.02	1.00–1.03	.037*
LVEF on echocardiography	0.99	0.96–1.01	.217			
NAC staging system			< .001*			●
Stage 2 vs 1 Stage 3 vs 1	2.31 4.03	1.18–4.54 1.84–8.86	.015* < .001*			
Mayo staging system			.001*			●
Stage 2 vs 1 Stage 3 vs 1	1.48 4.07	0.70–3.15 1.86–8.88	.306 < .001*			
Bone scintigraphy						
Heart/rib ratio (per 1) Heart/WB ratio (per 1) Elbow/rib ratio (per 1)	1.02 1.08 0.48	0.58–1.81 0.86–1.36 0.14–1.59	.942 .495 .154			● ● ●
Soft tissue/rib ratio (per 0.1)	1.31	0.97–1.78	.081	1.41	1.02–1.94	.038*
Shoulder/rib ratio (per 1) Wrist/rib ratio (per 1)	0.60 0.91	0.23–1.53 0.28–2.91	.281 .868			● ●

*ATTRwt* wild type transthyretin amyloid, *HR* hazard ratio, *95% CI* 95% confidence interval, *eGFR* estimated glomerular filtration rate, *NT-proBNP* N-terminal brain natriuretic propeptide, *hs-cTnT* high-sensitivity cardiac troponin T, *LVEF* left ventricular ejection fraction, *NAC* UK National Amyloidosis Centre, *H/WB* Heart-to-whole body, * = significant variable, ● = not tested

To evaluate whether soft tissue/rib ratio is independently associated with all-cause mortality besides two currently used staging models, soft tissue/rib ratio was added to multivariable models for both staging systems corrected for age [18, 19]. Results are presented in Table 3.

**Table 3:** Multivariable cox proportional hazards models for all-cause mortality in ATTRwt amyloidosis patients

**Model 1** **NAC staging + age + soft tissue/rib ratio**			
Variable	HR	95% CI	*p*-value
Age (per year)	1.03	0.98–1.09	.258
Soft tissue/rib ratio (per 0.1)	1.46	1.06–2,00	.021*
NAC stages			< .001*
Stage 2 vs 1 Stage 3 vs 1	2.28 5.07	1.15–4.53 2.22–11.60	.019* < .001*
**Model 2** **Mayo staging system + age + soft tissue/rib ratio**			
Variable	HR	95% CI	*p*-value
Age (per year)	1.02	0.97–1.08	.432
Soft tissue/rib ratio (per 0.1)	1.34	1.00–1.80	.053
Mayo stages			< .001*
Stage 2 vs 1 Stage 3 vs 1	1.43 4.09	0.67–3.04 1.88–8.89	.352 < .001*

*ATTRwt* wild type transthyretin, *HR* hazard ratio, *95% CI* 95% confidence interval, *NAC* UK National Amyloidosis Centre, * = significant variable

In the first model, the NAC staging system and soft tissue/rib ratio were both significantly associated with all-cause mortality, whereas age was not. The hazard ratio (HR) of the soft tissue/rib ratio was 1.46 (95% CI: 1.06–2.00, *p* = 0.021) for 0.1 point increase in soft tissue/rib ratio. In the second model, the Mayo staging system was significantly associated with all-cause mortality whereas age and soft tissue/rib ratio were not (soft tissue/rib ratio: HR: 1.34, 95% CI: 1.00–1.80, *p* = 0.053).

A total of 37 deaths (39%) were due to cardiac events and occurred after a median follow-up duration of 54 (95% CI 48–59) months. The cause of death was uncertain in two patients (2%), and these patients were excluded from the analysis. NT-proBNP, hs-cTnT, the NAC and Mayo prognostic staging systems and soft tissue/rib ratio were associated with cardiac mortality in univariable analysis. In a multivariable model containing NT-proBNP, hs-cTnT, age and soft tissue/rib ratio, hs-cTnT and soft tissue/rib ratio were the only variables independently associated with cardiac mortality. Soft tissue/rib ratio was independently associated with cardiac mortality alongside both prognostic staging systems. Hazard ratios and *p*-values are shown in online resources 1 and 2.

## Discussion

This study shows increased radiotracer uptake on bone scintigraphy in the heart and soft tissue of ATTRwt amyloidosis patients compared with control patients with non-amyloid heart failure. Furthermore, intensity of [^99m^Tc]Tc-HDP uptake in soft tissue on bone scintigraphy corresponds with the Congo red score in fat tissue aspirates in ATTRwt amyloidosis patients. Lastly, [^99m^Tc]Tc-HDP uptake in soft tissue was independently associated with all-cause and cardiac mortality in ATTRwt amyloidosis patients, even next to currently used prognostic staging models [18, 19]. Radiotracer uptake in soft tissue could therefore be an additional prognostic marker in ATTRwt amyloidosis patients.

The novel finding that soft tissue/rib ratio on bone scintigraphy corresponds with the Congo red score in fat tissue aspirates indicates that intensity of radiotracer uptake reflects amyloid load in soft tissue in ATTRwt amyloidosis patients. This is in line with a recent study showing that cardiac radiotracer uptake is associated with myocardial amyloid load [5]. The intensity of cardiac radiotracer uptake was not associated with amyloid load in fat tissue, likely due to non-homogeneous distribution of amyloid throughout the body [21].

Furthermore, we found that the intensity of radiotracer uptake in soft tissue is independently associated with all-cause and cardiac mortality in ATTRwt amyloidosis patients, even in multivariable models that include known covariates of mortality and two well-validated prognostic staging systems [18, 19]. Higher soft tissue/rib ratios are associated with poorer survival, likely attributable to increased amyloid burden due to advanced disease or more systemic distribution of amyloid throughout the body. This finding is consistent with a previous study by Malka et al. [7], associating worse survival with the presence of extracardiac radiotracer uptake in addition to cardiac uptake on bone scintigraphy. However, in contrast to the study of Malka et al., our study assessed the association of soft tissue radiotracer uptake with mortality independently from cardiac radiotracer uptake on bone scintigraphy and additionally took into account the intensity of radiotracer uptake.

Cardiac radiotracer uptake intensity on bone scintigraphy, expressed as continuous variable, was not associated with mortality, which is in line with previous studies [10, 11, 22]. One study reported a correlation between H/WB ratio on a continuous scale and major adverse cardiac events in a cohort consisting of hereditary ATTR amyloidosis patients with and without cardiomyopathy [12]. Given the evident difference in prognosis between patients with and patients without cardiomyopathy, it is unsurprising that this correlation was found in their study [12]. However, if the association of cardiac tracer uptake with mortality is studied within a cohort comprising patients with high grade cardiac radiotracer uptake on bone scintigraphy (Perugini grade 2 and 3), the range in cardiac radiotracer uptake, and thus cardiac amyloid load, may not be sufficient to find an association with outcomes [10, 11, 22].

In this study, extracardiac radiotracer uptake at various sites was compared between ATTRwt amyloidosis patients and non-amyloid heart failure controls. We expected to find increased radiotracer uptake in the shoulders and wrists of ATTRwt amyloidosis patients compared to non-amyloid heart failure patients, as increased incidence of shoulder pathology and presence of amyloid deposits in various tendons and the carpal tunnel have been described in these patients [23–25]. The lack of difference between the groups may result from the limited area of amyloid-containing structures (carpal tunnel and joint space) within a specific ROI. Additionally, increased radiotracer uptake in joints can occur in osteoarthritis, prevalent in individuals of age [26]. Since the median age of participants in this study is > 70 years, increased radiotracer uptake due to osteoarthritis might overshadow the potential increase caused by amyloid deposits in small structures within the ROIs.

We used a semi-quantitative ratio based on the anterior planar scintigraphy image, a common approach for evaluation of cardiac radiotracer uptake [17, 23, 24, 27]. Heart-to-contralateral lung and heart-to-whole-body ratios are commonly used ratios in cardiac amyloidosis, but are unsuitable for the assessment of soft tissue radiotracer uptake. Therefore, we used a rib as a reference tissue, as radiotracer uptake in bone tissue is likely to be more stable than in other tissues, despite potential competitive radiotracer uptake in advanced disease [28].

### Limitations

The main limitation of this study is using planar bone scintigraphy instead of SPECT images, primarily due to the absence of whole body SPECT scans and routine prospective SPECT-camera calibration. However, the use of planar images is in line with methods used in regular clinical practice, ensuring that results are easily translated into daily practice.

Additionally, the study's limited sample size increases the risk of falsely concluding that certain variables are not linked to mortality in ATTR-CM patients. This is particularly relevant for variables with *p*-values slightly above 0.05, like the soft tissue/rib ratio in univariable analysis and in the multivariable analysis including age and the Mayo staging system. A larger sample size might have revealed significant associations for these variables.

Furthermore, we pooled patients with Congo red-stained fat tissues graded 2–4 + . Although comparing the median soft tissue/rib ratio among all Congo red grades would have been interesting, it is noteworthy that high Congo red grades are uncommon in ATTRwt amyloidosis patients. The high percentage of positive fat tissue samples in our cohort, compared to previous reports [29], suggest careful analysis, ensuring precise examination of the association between soft tissue/rib ratios and amyloid load in our study.

### Implications

Our study suggests that bone scintigraphy contains more relevant information than typically extracted from the images. Further, radiotracer uptake in soft tissue on bone scintigraphy may serve as a surrogate of soft tissue amyloid load. If larger validation studies confirm that soft tissue radiotracer uptake is associated with mortality, it could enhance prognostic stratification for patients with ATTRwt amyloidosis and lead to more effective utilization of costly therapies.

### Conclusion

On planar bone scintigraphy, ATTRwt amyloidosis patients showed higher radiotracer uptake in the heart and in soft tissue compared with non-amyloid heart failure patients. The soft tissue/rib ratio on anterior planar images increased with increasing grades of Congo red-stained fat tissue samples and was independently associated with all-cause and cardiac mortality in ATTRwt amyloidosis patients. Soft tissue/rib ratio may serve as a new non-invasive and hands-on imaging marker of disease severity and prognosis. Further investigation of soft tissue radiotracer uptake on bone scintigraphy using other radiotracers and in larger cohorts, preferably using calibrated SPECT scans, is warranted.

## Supplementary Information

Below is the link to the electronic supplementary material.

Supplementary file1 (DOCX 24 KB)

## Data Availability

The datasets generated during and/or analysed during the current study are available from the corresponding author on reasonable request.

## Abbreviations

ATTR: Transthyretin amyloid
ATTRwt: Wild type transthyretin amyloid
ATTR-CM: ATTR-related cardiomyopathy
CI: Confidence interval
eGFR: Estimated glomerular filtration rate
HDP: Hydroxydiphosphonate
HR: Hazard ratio
hs-cTnT: High-sensitivity cardiac troponin T
H/WB: Heart-to-whole body
ICC: Intraclass correlation coefficient
LVEF: Left ventricular ejection fraction
NAC: UK National Amyloidosis Centre
NT-proBNP: N-terminal pro-brain-type natriuretic peptide
ROI: Region-of-interest
SPECT: Single photon emission computed tomography
TTR: Transthyretin
