# Supplementary material for: [99mTc]Tc-hydroxydiphosphonate uptake in soft tissue is associated with amyloid load in subcutaneous abdominal fat tissue and mortality in wild-type transthyretin amyloidosis patients
Source: Eur J Nucl Med Mol Imaging. 2024 Aug 8;52(1):88–97. doi: 10.1007/s00259-024-06865-w (PMC11599362; doi:10.1007/s00259-024-06865-w)
Supplement: Supplementary file 1 — Supplementary file1 (DOCX 24 KB) [file 259_2024_6865_MOESM1_ESM.docx]

**[^99m^Tc]Tc-hydroxydiphosphonate Uptake in Soft Tissue is Associated with Amyloid Load in Subcutaneous Abdominal Fat Tissue and Mortality in Wild-Type Transthyretin Amyloidosis Patients**

Tingen HSA^1^, Groothof D^2^, Tubben A^3^, Bijzet J^4^, Houwerzijl EJ^2^, Muntinghe FLH^2^, van der Zwaag PA^5^, van der Meer P^3^, Hazenberg BPC^6^, Slart RHJA^1,7^, Nienhuis HLA^2^

Departments of Nuclear Medicine and Molecular Imaging^1^, Internal Medicine^2^, Cardiology^3^, Laboratory Medicine^4^, Clinical Genetics^5^, and Rheumatology & Clinical Immunology^6^, Groningen Amyloidosis Center of Expertise, University Medical Center Groningen, Groningen, The Netherlands. Biomedical Photonic Imaging Group, Faculty of Science and Technology, University of Twente, Enschede, The Netherland^7^

**European Journal of Nuclear Medicine and Molecular Imaging**

*Corresponding author: H.S.A. Tingen,* [*h.s.a.tingen@umcg.nl*](mailto:h.s.a.tingen@umcg.nl)

***Online resource 1:*** Univariable and multivariable cox proportional hazards regression analysis for cardiac mortality in ATTRwt amyloidosis patients

| **Variable** | **HR** | **95% CI** | **p-value** | **HR** | **95% CI** | **p-value** |
| --- | --- | --- | --- | --- | --- | --- |
| **Age (per year)** | 1.00 | 0.95-1.06 | .943 | 1.01 | 0.95-1.08 | .739 |
| **Sex (male)** | 0.34 | 0.08-1.45 | .143 |  |  | ● |
| **Congo red grade** 1+ vs 0+  >2+ vs 0+ | 1.26  1.01 | 0.59-2.71  0.42-2.42 | .802  .555  .979 |  |  | ● |
| **Perugini score (grade 2)** | 1.09 | 0.56-2.12 | .793 |  |  | ● |
| **eGFR (per ml/min*1.73m^2^)** | 0.98 | 0.96-1.00 | .067 |  |  | ● |
| **NT-proBNP (per 100 ng/L)** | 1.01 | 1.01-1.02 | <.001* | 1.01 | 1.00-1.02 | .327 |
| **Hs-cTnT (per 1 ng/L)** | 1.02 | 1.01-1.03 | <.001* | 1.02 | 1.00-1.03 | .048* |
| **LVEF on echocardiography** | 0.98 | 0.95-1.01 | .189 |  |  | ● |
| **NAC staging system** Stage 2 vs 1  Stage 3 vs 1 | 1.87  3.01 | 0.86-4.08  1.22-7.46 | .044*  .114  .017* |  |  | ● |
| **Mayo staging system**  Stage 2 vs 1  Stage 3 vs 1 | 1.72  2.85 | 0.76-3.88  1.18-6.89 | .067*  .195  .020* |  |  | ● |
| **Bone scintigraphy**  Heart/rib ratio (per 1)  Heart/WB ratio (per 1)  Elbow/rib ratio (per 1)  Soft tissue/rib ratio (per 0.1)  Shoulder/rib ratio (per 1)  Wrist/rib ratio (per 1) | 1.17  1.15  0.51  1.45  0.79  0.53 | 0.59-2.31  0.88-1.49  0.13-2.02  1.04-2.01  0.28-2.18  0.14-2.11 | .657  .321  .341  .029*  .642  .372 | 1.48 | 1.05-2.09 | ●  ●  ●  .026*  ●  ● |
| ATTRwt = wild type transthyretin amyloid, HR = hazard ratio, 95% CI = 95% confidence interval, eGFR = estimated glomerular filtration rate, NT-proBNP = N-terminal brain natriuretic propeptide, hs-cTnT = high-sensitivity cardiac troponin T, LVEF = left ventricular ejection fraction, NAC = UK National Amyloidosis Centre, H/WB = Heart-to-whole body, * = significant variable, ● = not tested | | | | | | |

| ***Online resource 2:*** Multivariable cox proportional hazards models for cardiac mortality in ATTRwt amyloidosis patients | | | |
| --- | --- | --- | --- |
| **Model 1**  NAC staging + age + soft tissue/rib ratio | | | |
| **Variable** | **HR** | **95% CI** | **p-value** |
| ***Age (per year)*** | 1.01 | 0.95-1.07 | .794 |
| ***Soft tissue/rib ratio (per 0.1)*** | 1.56 | 1.10-2.20 | .012* |
| ***NAC stages  Stage 2 vs 1  Stage 3 vs 1*** | 1.74  3.94 | 0.79-3.86  1.52-10.22 | .017*  .171  .005* |
|  |  |  |  |
| **Model 2**  Mayo staging system + age + soft tissue/rib ratio | | | |
| **Variable** | **HR** | **95% CI** | **p-value** |
| ***Age (per year)*** | 1.01 | 0.95-1.07 | .800 |
| ***Soft tissue/rib ratio (per 0.1)*** | 1.44 | 1.04-1.99 | .027* |
| ***Mayo stages***  **Stage 2 vs 1**  **Stage 3 vs 1** | 1.66  2.90 | 0.73-3.77  1.20-7.00 | .061  .224  .018* |
| ATTRwt = wild type transthyretin, HR = hazard ratio, 95% CI = 95% confidence interval, NAC = UK National Amyloidosis Centre, * = significant variable | | | |
